# TS-LCD: Two-Stage Loop-Closure Detection Based on Heterogeneous Data Fusion

**DOI:** 10.3390/s24123702

**Published:** 2024-06-07

**Authors:** Fangdi Jiang, Wanqiu Wang, Hongru You, Shuhang Jiang, Xin Meng, Jonghyuk Kim, Shifeng Wang

**Affiliations:** 1 School of Optoelectronic Engineering, Changchun University of Science and Technology, Changchun 130022, China; jiangfangdi@outlook.com (F.J.); wangwanqiu0524@163.com (W.W.); youbinglian888@126.com (H.Y.); jiang_shuhang@163.com (S.J.); mengxin126536@163.com (X.M.); 2 Center of Excellence in Cybercrimes and Digital Forensics, Naif Arab University for Security Sciences, Riyadh 11452, Saudi Arabia; jkim@nauss.edu.sa; 3 Zhongshan Institute of Changchun University of Science and Technology, Zhongshan 528400, China

**Keywords:** loop-closure detection, multi-sensor fusion, timestamp synchronization, feature extraction

## Abstract

Loop-closure detection plays a pivotal role in simultaneous localization and mapping (SLAM). It serves to minimize cumulative errors and ensure the overall consistency of the generated map. This paper introduces a multi-sensor fusion-based loop-closure detection scheme (TS-LCD) to address the challenges of low robustness and inaccurate loop-closure detection encountered in single-sensor systems under varying lighting conditions and structurally similar environments. Our method comprises two innovative components: a timestamp synchronization method based on data processing and interpolation, and a two-order loop-closure detection scheme based on the fusion validation of visual and laser loops. Experimental results on the publicly available KITTI dataset reveal that the proposed method outperforms baseline algorithms, achieving a significant average reduction of 2.76% in the trajectory error (TE) and a notable decrease of 1.381 m per 100 m in the relative error (RE). Furthermore, it boosts loop-closure detection efficiency by an average of 15.5%, thereby effectively enhancing the positioning accuracy of odometry.

## 1. Introduction

Loop-closure detection has emerged as a promising approach to addressing the challenges encountered in simultaneous localization and mapping (SLAM) [1] technology. This technique, which holds a pivotal position in autonomous driving, augmented reality, and virtual reality, effectively mitigates the accumulation of errors in localization and map construction. By establishing robust constraints between the current frame and historical frames, loop-closure detection significantly enhances the practical utility of SLAM in autonomous navigation [2] and robotics applications [3]. Consequently, it facilitates the attainment of more precise and reliable spatial perception and navigation capabilities, thereby playing a pivotal role in ensuring the accuracy and efficiency of SLAM systems. Depending on the sensor employed, this methodology can be segmented into two primary categories: vision-based and laser-based loop-closure detection [4].

Vision-based loop-closure detection techniques hinge on visual features extracted from the surrounding environment. These features predominantly originate from scenes replete with textures, such as the facades of buildings or road signage [5,6,7]. Through the utilization of methodologies like bag-of-words modeling, the system possesses the capability to swiftly identify candidate loop-closure frames bearing similarities to the present scene amidst an extensive corpus of imagery [8,9]. Nevertheless, vision-based loop-closure detection exhibits notable sensitivity to environmental alterations. For instance, when a robot revisits a locale under varying lighting conditions or from altered perspectives, the system might fail to precisely recognize the loop closure due to substantial shifts in visual features. This ultimately yields erroneous detection outcomes [10]. Laser-based loop-closure detection methodologies exhibit enhanced robustness. These approaches generally involve the extraction of local or global descriptors from point clouds acquired through LiDAR scans. These descriptors remain unaffected by variations in illumination and viewing angles, thereby rendering them resilient to environmental changes. Nonetheless, laser-based loop-closure detection faces challenges in structurally homologous environments [11], such as elongated corridors or repetitive architectural layouts. In such scenarios, the system might erroneously identify distinct positions as loop closures due to the descriptor similarities, ultimately causing disarray within the navigation system and compromising localization accuracy.

**Figure 1:**
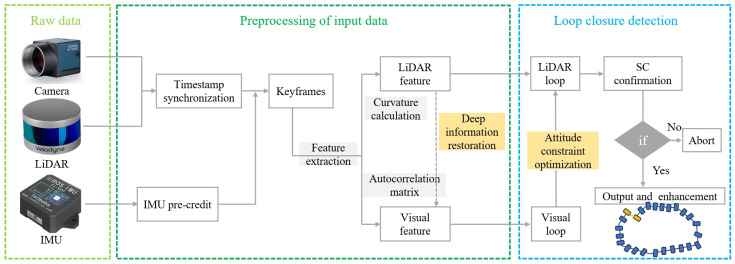
TS-LCD processing pipeline showing the LiDAR, camera, and IMU data input, preprocessing, and feature extraction. The loop-closure detection module utilizes both visual and LiDAR information for two-stage detection.

To address the prevalent issues and challenges associated with vision- and laser-based loop-closure detection, this paper presents an innovative loop-closure detection algorithm based on the principle of multivariate heterogeneous data fusion. Its algorithmic framework is shown in Figure 1. The present study overcomes the inherent performance limitations of single-sensor data in specific environments. Our approach harnesses the complementary strengths of multiple sensor data to enhance the accuracy and robustness of closed-loop inspection. The primary contributions of this study can be summarized as follows:

(1) This paper proposes an adaptive tightly coupled framework for loop-closure detection, named two-stage loop-closure detection (TS-LCD), achieving improved robustness and accuracy for closed-loop detection in different environments.
(2) An innovative method based on the interpolation technique is proposed in this paper, which optimizes the data processing flow and achieves timestamp synchronization.
(3) The effectiveness of the algorithm is validated through the integration of mainstream laser odometry frameworks and rigorous evaluation utilizing the KITTI dataset.

## 2. Related Works

Loop-closure detection holds significant importance in the domain of SLAM and has garnered considerable research attention in recent times. References [12,13,14] offer diverse algorithms for loop detection and location identification, each boasting unique strengths in efficiency, precision, and versatility. Two loop-closure detection approaches that are pertinent to our study are those reliant on vision and laser.

### 2.1. LiDAR-Based Loop-Closure Detection

Loop-closure detection methods for laser sensors can be categorized into local feature-based methods and global feature-based methods. For methods based on local feature fusion to obtain global features, Bosse et al. [15] divided the point cloud data into regions according to cylindrical shape and fused the average height, variance, overall roundness, and cylindricality of the point cloud in each region to obtain the global descriptor of the point cloud. Steder et al. [16] used the NARF features [17] as the local features and generated a bag-of-words model through the bag-of-words vectors for keyframe retrieval. Zaganidis et al. [18] used semantic segmentation to assist loop-closure detection for NDT statistical maps. For the global descriptor approach, Granström et al. [19] utilized point cloud rotational invariance, The authors used the statistical features of the point cloud as the parameters of the global descriptor, such as the point cloud range, volume, etc., and AdaBoost as the classifier of the features to complete the loop-closure detection. LeGO-LOAM added a loop-closure detection module based on the LOAM system and used the construction of a KD tree for the keyframe positions to search for the closest keyframe in the spatial location. Kim et al. [20] used Scan Context as the point cloud descriptor for loop-closure detection based on LeGO-LOAM. Lin et al. [21] proposed a new SLAM system that calculated the feature distribution of keyframes using a 2D statistical histogram for loop-closure detection. CNNs (Convolutional Neural Networks) have shown advantages in feature extraction, so many extraction methods have adopted CNN features. Yang et al. [22] used the PointNetVLAD network as a local feature extraction network for loop-closure detection. They improved the classification part of PointNetVLAD, trained the classification model for the extracted descriptors, and used cross-entropy with stochastic gradient descent as a loss function to improve the classification results of PointNetVLAD. Yin et al. [23] utilized the neural network LocNet to extract the global descriptors of point cloud frames and added loop-closure detection to the SLAM system based on the Monte Carlo localization algorithm. Zhu et al. [24] proposed the GOSMatch method, which employs semantic hierarchy descriptors and geometric constraints for loop-closure detection. They utilized RangeNet++ to detect the semantic information of the current frame of point cloud data, employed a statistical histogram of semantic object connectivity relations as the global descriptor of the semantic hierarchy of point cloud frames, and finally utilized the RANSAC algorithm for geometric validation. Vidanapathirana et al. [25] proposed the fusion of point cloud features and a point cloud frame spatio-temporal feature network called Locus for extracting global descriptors. The OverlapNet method [26] used pairs of depth maps, normal vector maps, intensity maps, and semantic-type maps of the point cloud to extract the global descriptors.

To address the lack of color and textural information in point cloud data, Zhu et al. [27] embedded a visual sensor-based loop-closure detection method into a laser SLAM system and proposed using an ORB-based bag-of-words model and the RANSAC algorithm to accomplish loop-closure detection. Krispel et al. [28] proposed a global feature extraction method that utilized fused image and point cloud features. Xie et al. [29] fused point cloud and image global descriptor extraction methods based on PointNetVLAD, utilizing PointNetVLAD and ResNet50 as the feature extraction methods for point cloud features and image features, respectively, and finally obtained global descriptors by fusing these features.

### 2.2. Vision-Based Loop-Closure Detection

The traditional VSLAM (visual simultaneous localization and mapping) system constructs the global descriptor of the current frame by extracting local manual features to complete the retrieval of loop-closure candidate frames. The loop-closure detection module of the ORB-SLAM system [30] adopts ORB feature points as the manual features and uses the bag-of-words model to construct the bag-of-words vector and complete the matching of candidate frames. Due to the weak robustness of manual features, which were easily affected by lighting, Zhang et al. [31] used a CNN to extract local features instead of manual features. Yue et al. [32] proposed adding the spatial structure information of the feature points and using triangular segmentation and graph validation as geometric constraints based on the extraction of local features using a CNN. However, the above methods cannot obtain the semantic information or dynamic and static attributes of the feature points.

For visual loop-closure detection in dynamic environments, Wang et al. [33] constructed a SURF feature database of dynamic objects offline and judged the motion attributes of the feature points based on the database. Migliore et al. [34] filtered static feature points through triangulation, and Mousavian et al. [35] used semantic segmentation to eliminate dynamic feature points, improving dynamic feature point recognition accuracy. Similarly, in DynaSLAM [36], a dynamic feature point rejection part was added to the ORB-SLAM2 system, rejecting dynamic object feature points through semantic segmentation based on MaskRCNN [37] and feature point geometric constraint relations. The authors also discussed the effects of adding image restoration to the SLAM system based on the rejection of dynamic feature points. In a related study on scene recognition, NetVLAD [38] improved VLAD by integrating local features to obtain global descriptors. It was successfully introduced into deep learning models, which could be trained to obtain global descriptors through deep learning networks. In addition, the researchers of CALC2.0 [39] designed a CNN-based approach that integrated appearance, semantic, and geometric information, categorizing all dynamic objects as “other” semantic attributes in terms of semantic labels. Although the dynamic objects were unified into the “other” category in CALC2.0, the method of generating global descriptors through deep learning networks was still affected by dynamic region pixels to varying degrees due to the lack of image preprocessing. To avoid the impact of dynamic scenes on the construction of global descriptors, Naseer et al. [40] used a CNN to segment the image and then extracted the global descriptors from the segmented image. Munoz et al. [41] used a network of object recognition methods instead of a segmentation network to modify the global descriptors of an image.

### 2.3. Deep Learning-Based Loop-Closure Detection

With the development of deep learning technology, learned local features have been employed for geometrical verification in LCD (loop-closure detection). Noh et al. [42] introduced the DEep Local Feature (DELF) approach, focusing on extracting and selectively utilizing local features via an attention mechanism tailored for geometrical verification. An et al. [43] presented FILD++, an innovative LCD system that leverages a two-pass CNN model to extract both global and local representations from input images. The geometrical verification between query and candidate pairs is subsequently conducted based on the locally learned features extracted through this process. Hausler et al. [44] proposed the patch-level feature approach (SaliencyNetVLAD), which further optimizes the pixel-level local features to cover a larger spatial range. Jin et al. [45] utilized the SaliencyNetVLAD method with a newly designed facet descriptor loss, enabling SaliencyNetVLAD to extract more discriminative facet-level local features. Jin et al. [46] proposed a generalized framework called LRN-LCD, a lightweight relational network for LCDs, which integrates the feature extraction module and the similarity measure module into a simple lightweight network.

Deep learning-based loop-closure detection possesses powerful feature extraction capabilities compared to traditional methods. Deep learning models are able to extract high-level features from complex images or point cloud data. These features are then compared to determine whether a loop closure has occurred. However, it still encounters some challenges in practical applications. The training and inference of deep learning models necessitate a significant amount of computational resources, posing a challenge for resource-limited devices. In this paper, we propose a two-stage loop-closure detection mechanism. The accuracy and robustness of closed-loop detection are improved by utilizing the complementary advantages of multi-sensor data.

## 3. Method

**Figure 2:**
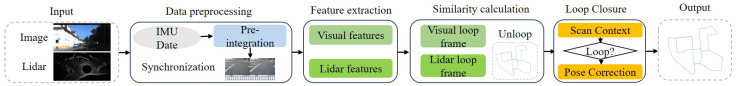
First, timestamp synchronization is performed. Then, feature extraction is performed to compute the similarity to obtain the selected frame after loop-closure detection, which is then further confirmed by SC.

The proposed TS-LCD algorithm framework is illustrated in Figure 2. The input consists of LiDAR point cloud and camera image data, which undergo parallel preprocessing to extract real-time LiDAR and visual features. The extracted features are stored in a local map. Subsequently, by preprocessing the IMU data, the pose information of keyframes is obtained. Further filtering and calibration of the IMU data can reduce the attitude error of the keyframes and improve the accuracy of visual loop-closure detection. In addition, IMU data can be used to address point cloud distortion arising from LiDAR movement. Finally, SC (Scan Context) is employed for loop-closure frame detection and matching to minimize false detection rates. The details of each module within the algorithm framework are introduced in order below.

### 3.1. Preprocessing of Input Data

Since the sampling frequencies of the LiDAR, camera, and IMU are not the same, this paper synchronizes the timestamps of the LiDAR and camera data by finding the nearest-neighbor frames based on data processing and interpolation. The position states of the LiDAR and camera keyframes are obtained by pre-integrating the IMU data.

#### 3.1.1. Timestamp Synchronization

**Figure 3:**
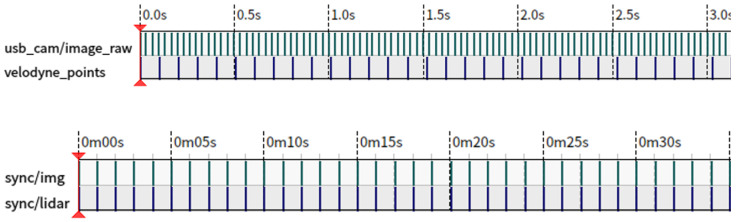
Timestamp synchronization. For data without timestamp synchronization, the camera sampling frequency is much larger than the LiDAR sampling frequency. After performing timestamp synchronization based on the sampling frequency of the LiDAR, the data stream of the camera is interpolated and synchronized, and finally, the synchronized timestamp is obtained at a frequency of 10 frames per second.

For each frame of the point cloud data, its timestamp $T_{L}$ is registered, and for each frame of the image, its timestamp $T_{C}$ is registered. The timestamps of the LiDAR and camera are sorted separately. For each LiDAR timestamp $T_{L_{i}}$, find the closest neighboring timestamps $T_{C_{j}}$ and $T_{C_{j+1}}$ in the camera timestamp sequence. If the neighboring camera timestamps $T_{C_{j}}$ and $T_{C_{j+1}}$ are located on either side of $T_{L_{i}}$, the camera timestamp corresponding to $T_{L_{i}}$ can be estimated using linear interpolation or other interpolation methods. The linear interpolation formula is:

(1) $$T_{C_{est}}=T_{C_{j}}+\left(T_{L_{i}}-T_{C_{j}}\right)\times \frac{T_{C_{j+1}}-T_{C_{j}}}{T_{L_{i+1}}-T_{L_{i}}}$$

where $T_{C_{est}}$ is the estimated camera timestamp corresponding to $T_{L_{i}}$. As shown in Figure 3.

#### 3.1.2. IMU Pre-Credit

**Figure 4:**
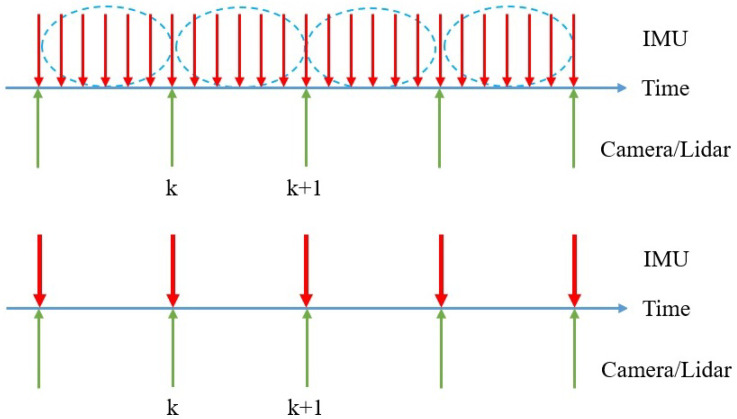
Pre-integration of IMU data for obtaining the pose of keyframes. The red arrow indicates IMU observation, and the green arrow indicates Camera and LiDAR observation.

The IMU acquires the acceleration and angular velocity, and the position information of the keyframes can be obtained through the integration operation of the IMU measurements. The sampling frequency of the IMU is much larger than the keyframe release frequency of the image and the laser, corresponding to the red line and the green line in Figure 4, respectively. Assuming that the two neighboring red lines correspond to moments *k* and k+1, the average acceleration and the average angular velocity of the IMU during this time period are, respectively,

(2) $${\bar{\hat{a}}}_{\dot{k}}=\frac{1}{2}\left[q_{\dot{k}}\left({\hat{a}}_{\dot{k}}-b_{a_{k}}\right)+q_{\dot{k}+1}\left({\hat{a}}_{\dot{k}+1}-b_{a_{k}}\right)\right]$$

(3) $$\bar{\hat{w_{k}}}=\frac{1}{2}\left(\hat{w_{k}}+\hat{w_{k+1}}\right)-b_{w_{k}}$$

where ${\hat{a}}_{k}$ and ${\hat{a}}_{k+1}$ are the accelerations of *k* and k+1, respectively; $b_{a_{k}}$ and $b_{w_{k}}$ are the zero-biases; $\hat{w_{k}}$ and $\hat{w_{k+1}}$ are the angular velocities of *k* and k+1, respectively; and $q_{k}$ and $q_{k+1}$ are the directional state quantities (DSPs) of *k* and k+1, respectively.

At moment k+1, the position ${\hat{\alpha }}_{k+1}^{b_{k}}$, velocity ${\hat{\beta }}_{k+1}^{b_{k}}$, and attitude ${\hat{\gamma }}_{k+1}^{b_{k}}$ of a keyframe can be expressed as follows:

(4) $${\hat{\alpha }}_{k+1}^{b_{k}}={\hat{\alpha }}_{k}^{b_{k}}+{\hat{\beta }}_{k}^{b_{k}}\delta t+\frac{1}{2}{\hat{\hat{\alpha }}}_{k}\delta t^{2}$$

(5) $${\hat{\beta }}_{k+1}^{b_{k}}={\hat{\beta }}_{\dot{k}}^{b_{k}}+{\bar{\hat{\hat{a}}}}_{\dot{k}}\delta t$$

(6) $${\hat{\gamma }}_{k+1}^{b_{k}}={\hat{\gamma }}_{k}^{b_{k}}⊗{\hat{\gamma }}_{k+1}^{k}={\hat{\gamma }}_{k}^{b_{k}}⊗\left[\frac{1}{\frac{1}{2}\bar{{\hat{w}}_{k}}\delta t}\right]$$

where δt is the time interval from frame *k* to frame k+1.

### 3.2. Feature Extraction

**Figure 5:**
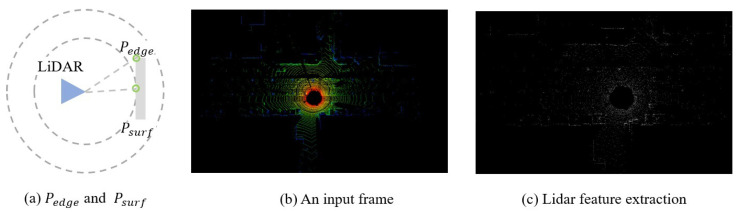
Each frame of the point cloud is subjected to feature extraction by calculating the curvature of each point; lower curvatures are defined as LiDAR planar features $P_{surf}$, and higher curvatures are defined as LiDAR edge features $P_{edge}$.

Assuming that the internal and external parameters of the LiDAR and camera are known and fixed, and their distortions have been corrected, in this paper, we adopt the curvature extraction method defined in LOAM to obtain LiDAR features. LiDAR features with higher curvatures are defined as LiDAR edge features $P_{edge}$, while those with lower curvatures are defined as LiDAR planar features $P_{surf}$. The LiDAR feature extraction method is shown in Figure 5. The curvature calculation formula is as follows:

(7) $$c=\frac{1}{|S|\cdot \|X_{\left(\dot{k},i\right)}^{L}\|}\|\sum_{j\in S,j\neq i}\left(X_{\left(\dot{k},i\right)}^{L}-X_{\left(\dot{k},j\right)}^{L}\right)\|$$

where S is the set of consecutive points returned by the laser in the same frame and $X_{\left(k,i\right)}^{L}$ and $X_{\left(k,j\right)}^{L}$ refer to the *i* and *k* points in the point cloud of the *k* scan in the *L* (LiDAR) coordinate system.

**Figure 6:**
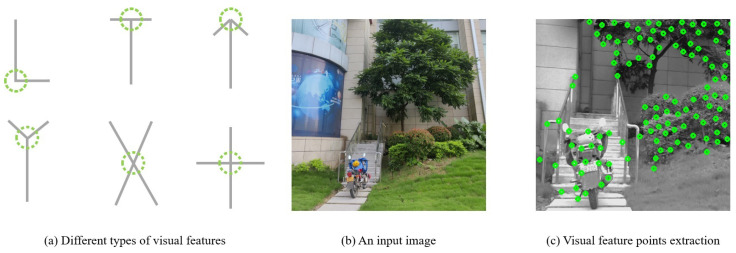
The image grayscale-based method detects visual features by calculating the curvature and gradient of the points.

For the selection of visual features, this paper calculates the autocorrelation matrix of each pixel point in the image and then determines whether the point is a corner point based on the eigenvalues of this matrix. Corner points are areas where the image signal changes significantly in two-dimensional space, which typically include significant change areas such as corner points, intersections, and textures. The visual feature extraction method is shown in Figure 6. The expression of the autocorrelation matrix M is

(8) $$M=[I_{x}^{2}I_{x}I_{y};\;I_{x}I_{y}I_{y}^{2}]$$

where $I_{x}$ and $I_{y}$ are the gradients of the image in the *x* and *y* directions, respectively.

### 3.3. Deep Information Correlation

**Figure 7:**
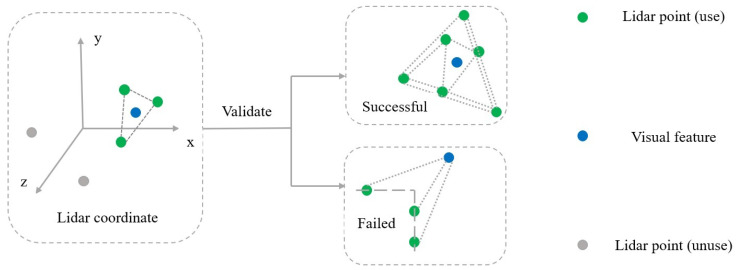
Deep information linkage framework process. The x, y and z axes are the coordinate system of LiDAR respectively.

Since a single camera does not have the ability to accurately measure depth information, only a scaled estimation of the feature point depth can be made, and the depth estimation will be highly noisy if the number of observations of the feature point is low or the parallax is insufficient. Within a multimodal sensor fusion framework, the depth information of visual feature points can be optimized using the LiDAR point cloud to improve the robustness and accuracy of the visual-inertial odometry. The depth information correlation module is designed to more accurately assign depths to visual features. As shown in Figure 7, visual features are projected into the LiDAR coordinate system through an external parameter matrix.

For each visual feature, the three closest LiDAR points can be selected via a KD-tree. Depending on the depth of these points, a validation process is performed to improve the accuracy of subsequent matching. The specific validation process involves calculating the Euclidean spatial distances of the three nearest LiDAR points to the current visual feature. In the experiment, if the farthest distance between the three points is less than 0.5 m, the validation is successful, and the depth can be calculated by bilinear interpolation. Otherwise, triangulation is applied to assign depths to visual features. Figure 7 shows the exact process of validation. The purpose of the validation process is to check whether the points are in the same plane; if the depth difference is too large, the points may be in different planes and therefore need to be excluded, thus reducing the possibility of false matches.

### 3.4. Loop-Closure Detection

The Dbow2 bag-of-words model is used as the basis of visual loop-closure detection in this paper. Firstly, the feature points in the latest keyframes tracked by optical flow are used to calculate the corresponding descriptors, which are then matched with the history frames to search for the most compatible frames and eliminate the history frames that are outside the time or distance thresholds with the current frame. Then, the attitude data of the remaining historical frames are obtained, and the position relationship between the current frame and the historical frames is optimized by the PnP (perspective-n-point) algorithm. In order to improve the accuracy of the loop-closure constraint, the attitude information of the current frame relative to the historical frames in the visual loop closure is sent to the LiDAR odometer as the initial value. The LiDAR odometer searches for the optimal match among the stored historical keyframes according to the minimum distance principle and then matches them according to the initial value provided by the visual loop closure to further optimize the attitude constraints, ultimately achieving high-precision loop-closure detection results.

**Figure 8:**
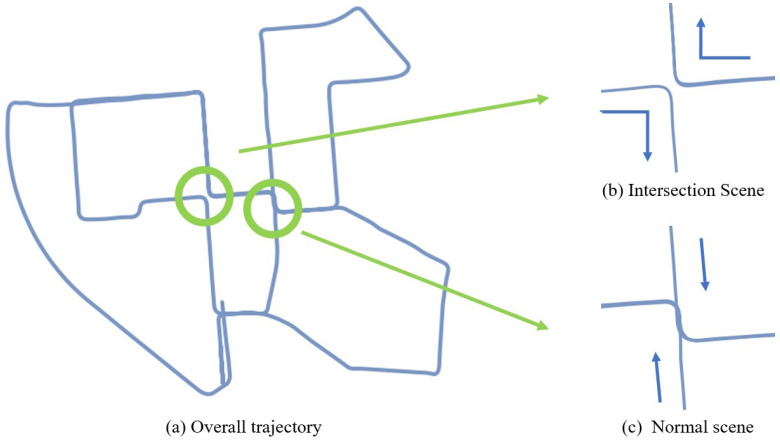
Failure of visual loop-closure detection. The arrow indicates the direction of travel of the vehicle.

In addition to the optimization of visual loop-closure detection from coarse to fine, to solve the defective view angle problem of visual loop-closure detection, as shown in Figure 8, a low-consumption LiDAR loop-closure detection mechanism is added based on visual loop-closure detection. The LiDAR odometer stores the position of historical keyframes in real time, and when it detects that the positional information of the point cloud in the latest frame is close to the historical trajectory, the loop-closure detection mechanism searches for the historical information and performs a match. Usually, when there is no visual loop-closure detection, this kind of LiDAR loop-closure detection method may fail due to the large scene. However, in this paper, we use the coupling of two types of loop-closure methods to address loop-closure detection in ordinary scenes. Through the visual loop closure, we aim to reduce the trajectory error under prolonged system operation. Meanwhile, the accuracy of LiDAR loop-closure detection based on spatial distance can be improved significantly to compensate for loop-closure failures caused by visual perspective problems. Additionally, we introduce the SC (Scan Context) algorithm to enhance the reliability and accuracy of loop-closure detection by precisely computing the degree of closed-loop recognition. The algorithmic framework is outlined below.
**Algorithm 1** Procedure of the proposed online solution  1:**Input**:The data sequence $\bar{U}$ after timestamp synchronization is divided into consecutive keyframes $\left(U_{1},U_{2},\ldots ,U_{K-1}\right)$, the paired observations $T={\left\{T_{C_{ex}}\right\}}_{T_{L}}^{T_{C}}$, linear interpolation (math.).  2:**Third-party libraries**:Kalman Filter KF; Random Sample Consensus (RANSAC); Scan Context (SC) loop-closure detection.  3:**Output**: The set of features for each frame of data $P_{edge}$, $P_{surf}$, and Harris Feature Point.  4:**Initialize**: Initialize keyframe database; OpenCV and PCL;  5:/* Visual loop-closure detection. */  6:**for** $\mathrm{New}\;\mathrm{keyframe}\;T_{C}\;\mathrm{in}\;\bar{U}$ **do**  7:   **if** rotationAngle > $60^{\circ }$ **then**  8:      Similarity S(A,B) Calculation by $S\left(A,B\right)=\frac{V_{A}\cdot V_{B}}{|V_{A}\left|\cdot \right|V_{B}|}$;  9:      **if** S(A,B) > 0.75 **then**         /* Output visual loopback candidate frames $T_{C}^{\mathrm{Loop}}$ = $T_{L}^{\mathrm{Loop}}$ */10:      **else** **if** S(A,B) < 0.75 **then**11:         Remove $T_{C}$;12:      **end if**13:   **end if** movingDistance > 10.0 h **then**14:      Similarity calculation module;15:      **if** S(A,B) > 0.75 **then**         /* Output visual loopback candidate frames $T_{C}^{\mathrm{Loop}}$ = $T_{L}^{\mathrm{Loop}}$ */16:      **else** **if** S(A,B) < 0.75 **then**17:         Remove $T_{C}$;18:      **end if**19:      Remove $T_{C}$;20:   **end if**21:**end for**22:/* Laser loop-closure detection. */23:/* Laser loop closure frame $T_{L}^{\mathrm{Loop}}$ extraction module similar to visual. */24:Similarity $dist(D_{1},D_{2})$ Calculation by $dist\left(D_{1},D_{2}\right)=\sqrt{\sum_{i=1}^{n}{\left(D_{1}\left[i\right]-D_{2}\left[i\right]\right)}^{2}}$;25:/* Loop-closure detection acknowledgment. */26:**for** Each pair of candidate loopback frames $T_{L}^{\mathrm{Loop}}$ and $T_{\mathrm{Correspond}}$ **do**27:   /* 3D matrix generation module for spatial structure information SC. */28:   **for** each SC **do**29:      /* Feature extraction module. */30:      /* Similarity calculation module. */31:      **if** Similarity meets the requirements **then**32:         /*Output loop-closure frames for optimization*/33:      **end if then**34:         Remove $T_{L}^{\mathrm{Loop}}$;35:      **end if**36:   **end for**37:**end for**

## 4. Experiments

The proposed TS-LCD framework was validated on the KITTI dataset, one of the largest publicly available datasets in the field of autonomous driving. The KITTI dataset comprises 11 sequences with ground-truth data (sequences 00–10), spanning a total length of 22 km and featuring a rich and diverse range of environments, including rural, urban, highway, and other mixed scenes. In this paper, we used datasets from sequences 00, 05, 06, and 07, which contain loop-closure data, for experimentation. To ensure a fair assessment of robustness and accuracy, the proposed framework was validated across all KITTI sequences with ground-truth data. The input data for these sequences consisted solely of LiDAR and image data, and the frequencies of the LiDAR and camera were pre-synchronized to 10 Hz using an algorithm. The root mean square error (RMSE) was calculated using EVO as an evaluation metric.

### 4.1. Evaluation of Odometer Positioning Accuracy

The experimental inputs consisted of binocular camera images, LiDAR point clouds, and IMU data from the KITTI dataset. The primary evaluation metric of the KITTI dataset was the average translational error (ATE), measured in terms of the drift per hundred meters and typically expressed as a percentage. The secondary metric was the average rotational error (ARE), measured in $\frac{\deg}{100\;m}$. LOAM is one of the best-performing LiDAR odometry algorithms on the KITTI benchmark, and SC is one of the most widely used loop-closure algorithms in laser SLAM applications. Therefore, in our experiments, we selected LOAM and SC-LOAM as baselines to validate the effectiveness of the proposed TS-LCD loop-closure framework. TS-LOAM denotes our algorithm combining TS-LCD and LOAM.

Odometry is an essential component of SLAM, and its accuracy is primarily reflected in the precision of trajectories without loops. Here, we compare the odometry performance of TS-LOAM with LOAM and SC-LOAM. The quantitative comparison results are shown in Table 1 and Table 2, where TS-LOAM refers to the addition of our loop-closure algorithm to LOAM. Compared to the baseline algorithms, TS-LOAM achieved the best performance, with an ATE of 1.87 and an ARE of 1.13 $\frac{\deg}{100\;m}$. The ATE was reduced by an average of 2.66 and the ARE by an average of 1.44 $\frac{\deg}{100\;m}$.

### 4.2. Comparison of Odometer Trajectory and Ground Truth

**Figure 9:**
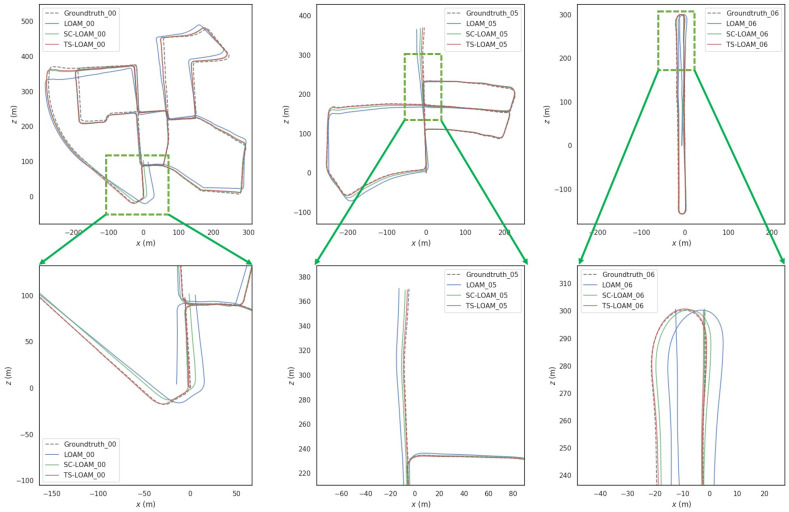
Plot of odometer trajectories versus ground truth using KITTI 00, 05, and 06 datasets. As seen in the figure, our proposed algorithm outperformed the two baseline algorithms in every position due to our second-order loop-closure matching mechanism.

To further analyze the advantages of the proposed framework, a qualitative analysis was conducted using sequences 00, 05, and 07. The results are shown in Figure 9. In these scenarios, TS-LOAM demonstrated superior performance compared to both LOAM and SC-LOAM. When compared to the ground-truth trajectory, the improved accuracy of TS-LOAM was evident on end-to-end drift constraints, primarily due to the second-order loop-closure detection strategy. The green rectangular boxes mark the areas where TS-LOAM showed significant improvements. Both LOAM and SC-LOAM exhibited noticeable drift, while TS-LOAM consistently maintained alignment with the ground-truth trajectory, proving the high robustness of the proposed algorithm.

### 4.3. Experimental Validation Using an Unmanned Vehicle

**Figure 10:**
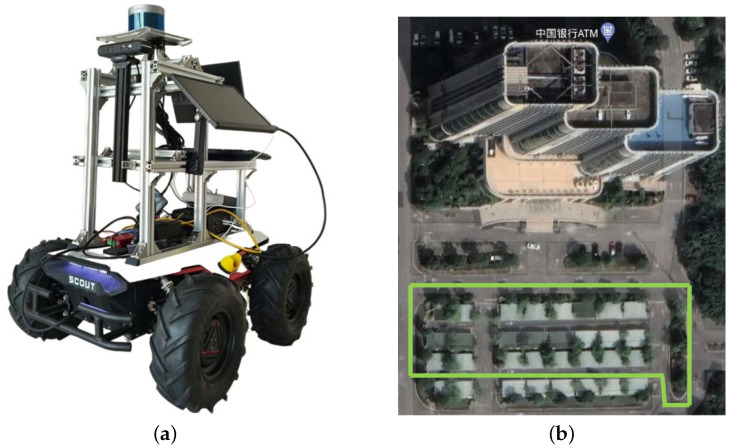
Unmanned vehicle experimental platform and experimental site. (**a**) Experimental platform; (**b**) Experimental site. The experimental site location is the Digital Building in Zhongshan City, China. The green color is the experimental track of driving.

The unmanned vehicle experiment used an unmanned vehicle equipped with a 16-line LiDAR (RS-Helios 16), a six-axis IMU (HFI-B6), and a binocular camera (Astra Pro). The experiment was conducted in a campus setting, with the experimental equipment shown in Figure 10a. We selected a circular route around the parking lot in the campus environment for experimental analysis. Figure 10b shows the projection of the traveling trajectory of the unmanned vehicle on the satellite map.

**Figure 11:**
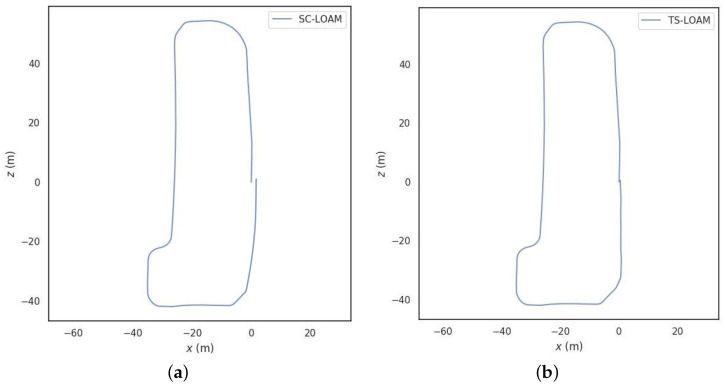
Comparison of trajectory information. (**a**) SC-LOAM trajectory; (**b**) TW-LOAM trajectory.

Figure 11a shows the data trajectory information from the SC-LOAM operational experiments. Failure to detect the loop closure while traveling the closed-loop section resulted in a shifted trajectory. The algorithm proposed in this paper (TS-LOAM) is shown in Figure 11b after integrating visual loop closure. It accurately detects the loop closure and optimizes the trajectory. By comparing the performance of the traditional SC-LOAM algorithm with the TS-LOAM algorithm proposed in this paper, the effectiveness of the two-stage loop-closure detection system in optimizing unmanned vehicle trajectory offsets is verified.

### 4.4. Loop-Closure Detection Performance

The performance of SLAM loop-closure detection is conventionally appraised by two primary metrics: precision and recall. Precision pertains to the proportion of genuinely detected loops among all the loops identified by the system, as exhibited in Table 3. In contrast, recall denotes the likelihood of a genuine loop being accurately detected within the system. The calculation formulas are as follows:

(9) $$Presion=\frac{TP}{TP+FP}$$

(10) $$Recall=\frac{TP}{TP+FN}$$

The loop-closure detection experiments used the publicly available dataset KITTI to evaluate the performance of loop-closure detection and compare it with the loop-closure results of SC-LOAM. The loop-closure detection scheme based on multi-sensor fusion exhibits higher robustness and can more accurately screen out the candidate loop-closure frames. As shown in Table 4, the algorithm improves the accuracy of loop-closure detection by 16.7% and recall by 14.3% relative to the SC-LOAM algorithm.

## 5. Conclusions

This paper proposes a framework for a loop-closure detection algorithm based on multi-sensor adaptive tight coupling, aiming to achieve accuracy and effectiveness in loop-closure detection. The proposed framework addresses the issue of mismatched visual loop frames and laser loop frames due to different sampling frequencies between LiDAR and cameras by utilizing data processing and interpolation techniques. Additionally, to enhance the accuracy of loop-closure detection, a second-order loop-closure detection scheme is introduced. To validate the robustness and accuracy of the proposed framework, extensive experiments are conducted on the KITTI dataset. The results demonstrate that compared to existing methods, the proposed TS-LCD and TS-LOAM framework significantly reduces the absolute translational error (ATE) by an average of 2.76% and the absolute rotational error (ARE) by 1.381 m/100 m. In addition, it improves closed-loop inspection efficiency by an average of 15.5%. In future work, we plan to incorporate deep neural networks for semantic segmentation of point cloud data within the existing algorithm framework and construct point cloud semantic graph descriptors using graph models.

## Figures and Tables

**Table 1:** Translational error index of odometry localization accuracy. Unit: %.

Algorithm	Seq. No.	Max.	Mean	Median	Min.	RMSE	Std.
LOAM	00	16.638040	6.509289	5.385290	1.038895	7.757144	4.219293
	05	12.416641	3.532057	3.166770	1.007233	4.095342	2.072775
	06	17.947513	7.720748	6.196751	0.000000	8.941244	4.509534
	07	1.511218	0.673462	0.676061	0.210234	0.708818	0.221072
	Avg	12.158353	4.608889	3.856218	0.564091	5.375637	2.755669
SC-LOAM	00	13.155136	3.725957	3.023545	0.752738	4.460252	2.451753
	05	3.872862	1.741009	1.572590	0.778893	1.891268	0.738771
	06	13.888364	6.792742	5.920660	0.000000	7.505593	3.192583
	07	1.639146	0.657002	0.678615	0.208712	0.700873	0.24407
	Avg	8.138877	3.229178	2.798853	0.435086	3.639497	1.656794
TS-LOAM	00	3.209416	1.317889	1.246500	0.296522	1.437708	0.574607
	05	2.788561	1.039795	0.937344	0.222877	1.153234	0.498775
	06	7.316305	3.818756	3.747921	0.000000	4.198256	1.744263
	07	1.182524	0.658270	0.638349	0.149834	0.683565	0.184231
	Avg	3.624202	1.708678	1.642529	0.167308	**1.868191**	0.750469

**Table 2:** Rotational error index of odometry localization accuracy. Unit: $\frac{\deg}{100\;m}$.

Algorithm	Seq. No.	Max.	Mean	Median	Min.	RMSE	Std.
LOAM	00	7.572816	2.861219	3.293702	0.052028	3.371129	1.782677
	05	5.962009	2.606564	3.083750	0.012628	3.052875	1.589298
	06	5.957210	2.001717	0.118190	0.015152	3.184903	2.477243
	07	6.893125	2.783081	3.128018	0.036888	3.337638	1.842359
	Avg	6.596290	2.563145	2.405915	0.029174	3.236637	1.922894
SC-LOAM	00	6.944201	1.683658	1.900435	0.026070	2.014907	1.106863
	05	4.717202	1.533127	1.627286	0.010259	1.824622	0.989327
	06	5.536863	1.086076	0.095882	0.014956	1.696411	1.303168
	07	5.404435	1.694663	1.642660	0.024214	2.054873	1.162161
	Avg	5.650675	1.499381	1.316566	0.018875	1.897703	1.140380
TS-LOAM	00	4.492446	1.024355	1.004043	0.004296	1.272371	0.754736
	05	3.267100	0.914836	1.012487	0.001205	1.110065	0.628745
	06	2.954673	0.913674	0.142899	0.007247	1.460142	1.138953
	07	1.182524	0.658270	0.638349	0.149834	0.683565	0.184231
	Avg	2.974185	0.877784	0.699445	0.040646	**1.131535**	0.676667

**Table 3:** Evaluation of loop-closure detection parameters.

Algorithm Judgment\Factual Truth Value	Be Looped	No Loop
Be looped	True Positive	False Positive
No loop	False Negative	True Negative

**Table 4:** Comparison of loop-closure detection results.

Dataset	SC-LOAM		TS-LOAM	
	Accuracy Rate	Recall Rate	Accuracy Rate	Recall Rate
00	5/5	5/7	6/6	6/7
Our data	0/1	0/1	1/1	1/1

## Data Availability

Data are contained within the article.

## References

[1] Taheri H., Xia Z.C. (2021). SLAM; definition and evolution. Eng. Appl. Artif. Intell..

[2] Ok K., Liu K., Frey K., How J.P., Roy N. Robust object-based slam for high-speed autonomous navigation. Proceedings of the 2019 International Conference on Robotics and Automation (ICRA).

[3] Yarovoi A., Cho Y.K. (2024). Review of simultaneous localization and mapping (SLAM) for construction robotics applications. Autom. Constr..

[4] Chen S., Zhou B., Jiang C., Xue W., Li Q. (2021). A LiDAR/visual slam backend with loop closure detection and graph optimization. Remote Sens..

[5] Wang C., Wu Z., Chen Y., Zhang W., Ke W., Xiong Z. (2023). Improving 3D Zebrafish Tracking with Multi-View Data Fusion and Global Association. IEEE Sens. J..

[6] Wang C., Wu Z., Ke W., Xiong Z. (2024). A simple transformer-based baseline for crowd tracking with Sequential Feature Aggregation and Hybrid Group Training. J. Vis. Commun. Image Represent..

[7] Wu Z., Wang C., Zhang W., Sun G., Ke W., Xiong Z. (2024). Online 3D behavioral tracking of aquatic model organism with a dual-camera system. Adv. Eng. Inform..

[8] Wang Y., Qiu Y., Cheng P., Duan X. (2020). Robust loop closure detection integrating visual–spatial–semantic information via topological graphs and CNN features. Remote Sens..

[9] Wang W., Liu J., Wang C., Luo B., Zhang C. (2021). DV-LOAM: Direct visual LiDAR odometry and mapping. Remote Sens..

[10] Artal R. (2017). Mur Real-Time Accurate Visual SLAM with Place Recognition. Ph.D. Thesis.

[11] Huang S., Dissanayake G. Convergence analysis for extended Kalman filter based SLAM. Proceedings of the 2006 IEEE International Conference on Robotics and Automation (ICRA 2006).

[12] Lowry S., Sünderhauf N., Newman P., Leonard J.J., Cox D., Corke P., Milford M.J. (2015). Visual place recognition: A survey. IEEE Trans. Robot..

[13] Masone C., Caputo B. (2021). A survey on deep visual place recognition. IEEE Access.

[14] Chen Y., Gan W., Zhang L., Liu C., Wang X. A survey on visual place recognition for mobile robots localization. Proceedings of the 2017 14th Web Information Systems and Applications Conference (WISA).

[15] Bosse M., Zlot R. Place recognition using keypoint voting in large 3D LiDAR datasets. Proceedings of the 2013 IEEE International Conference on Robotics and Automation.

[16] Steder B., Ruhnke M., Grzonka S., Burgard W. Place recognition in 3D scans using a combination of bag of words and point feature based relative pose estimation. Proceedings of the 2011 IEEE/RSJ International Conference on Intelligent Robots and Systems.

[17] Steder B., Rusu R.B., Konolige K., Burgard W. NARF: 3D range image features for object recognition. Proceedings of the Workshop on Defining and Solving Realistic Perception Problems in Personal Robotics at the IEEE/RSJ International Conference on Intelligent Robots and Systems, IROS 2020.

[18] Zaganidis A., Zerntev A., Duckett T., Cielniak G. Semantically Assisted Loop Closure in SLAM Using NDT Histograms. Proceedings of the 2019 IEEE/RSJ International Conference on Intelligent Robots and Systems (IROS).

[19] Granström K., Schön T.B., Nieto J.I., Ramos F.T. (2011). Learning to close loops from range data. Int. J. Robot. Res..

[20] Kim G., Kim A. Scan context: Egocentric spatial descriptor for place recognition within 3D point cloud map. Proceedings of the 2018 IEEE/RSJ International Conference on Intelligent Robots and Systems (IROS).

[21] Lin J., Zhang F. (2019). A fast, complete, point cloud based loop closure for LiDAR odometry and mapping. arXiv.

[22] Yang Y., Song S., Toth C. (2020). CNN-based place recognition technique for LIDAR SLAM. Int. Arch. Photogramm. Remote Sens. Spat. Inf. Sci..

[23] Yin H., Wang Y., Ding X., Tang L., Huang S., Xiong R. (2019). 3D LiDAR-based global localization using siamese neural network. IEEE Trans. Intell. Transp. Syst..

[24] Zhu Y., Ma Y., Chen L., Liu C., Ye M., Li L. GOSMatch: Graph-of-Semantics Matching for Detecting Loop Closures in 3D LiDAR data. Proceedings of the 2020 IEEE/RSJ International Conference on Intelligent Robots and Systems (IROS).

[25] Vidanapathirana K., Moghadam P., Harwood B., Zhao M., Sridharan S., Fookes C. Locus: LiDAR-based place recognition using spatiotemporal higher-order pooling. Proceedings of the 2021 IEEE International Conference on Robotics and Automation (ICRA).

[26] Chen X., Läbe T., Milioto A., Röhling T., Vysotska O., Haag A., Behley J., Stachniss C. (2021). OverlapNet: Loop closing for LiDAR-based SLAM. arXiv.

[27] Zhu Z., Yang S., Dai H., Li F. Loop Detection and Correction of 3D Laser-Based SLAM with Visual Information. Proceedings of the 31st International Conference on Computer Animation and Social Agents.

[28] Krispel G., Opitz M., Waltner G., Possegger H., Bischof H. Fuseseg: LiDAR point cloud segmentation fusing multi-modal data. Proceedings of the 2020 IEEE/CVF Winter Conference on Applications of Computer Vision.

[29] Xie S., Pan C., Peng Y., Liu K., Ying S. (2020). Large-scale place recognition based on camera-LiDAR fused descriptor. Sensors.

[30] Mur-Artal R., Montiel J.M.M., Tardos J.D. (2015). ORB-SLAM: A versatile and accurate monocular SLAM system. IEEE Trans. Robot..

[31] Zhang X., Su Y., Zhu X. Loop closure detection for visual SLAM systems using convolutional neural network. Proceedings of the 2017 23rd International Conference on Automation and Computing (ICAC).

[32] Yue H., Miao J., Yu Y., Chen W., Wen C. Robust Loop Closure Detection based on Bag of SuperPoints and Graph Verification. Proceedings of the 2019 IEEE/RSJ International Conference on Intelligent Robots and Systems (IROS).

[33] Wang Y.T., Lin M.C., Ju R.C. (2010). Visual SLAM and moving-object detection for a small-size humanoid robot. Int. J. Adv. Robot. Syst..

[34] Migliore D., Rigamonti R., Marzorati D., Matteucci M., Sorrenti D.G. Use a single camera for simultaneous localization and mapping with mobile object tracking in dynamic environments. Proceedings of the ICRA Workshop on Safe Navigation in Open and Dynamic Environments: Application to Autonomous Vehicles.

[35] Mousavian A., Košecká J., Lien J.M. Semantically guided location recognition for outdoors scenes. Proceedings of the 2015 IEEE International Conference on Robotics and Automation (ICRA).

[36] Bescos B., Fácil J.M., Civera J., Neira J. (2018). DynaSLAM: Tracking, mapping, and inpainting in dynamic scenes. IEEE Robot. Autom. Lett..

[37] He K., Gkioxari G., Dollár P., Girshick R. Mask R-CNN. Proceedings of the 2017 IEEE International Conference on Computer Vision (ICCV).

[38] Arandjelovic R., Gronat P., Torii A., Pajdla T., Sivic J. NetVLAD: CNN architecture for weakly supervised place recognition. Proceedings of the 2016 IEEE Conference on Computer Vision and Pattern Recognition (CVPR).

[39] Merrill N., Huang G. CALC2.0: Combining appearance, semantic and geometric information for robust and efficient visual loop closure. Proceedings of the 2019 IEEE/RSJ International Conference on Intelligent Robots and Systems (IROS).

[40] Naseer T., Oliveira G.L., Brox T., Burgard W. Semantics-aware visual localization under challenging perceptual conditions. Proceedings of the 2017 IEEE International Conference on Robotics and Automation (ICRA).

[41] Munoz J.P., Dexter S. (2020). Improving Place Recognition Using Dynamic Object Detection. arXiv.

[42] Noh H., Araujo A., Sim J., Weyand T., Han B. Large-scale image retrieval with attentive deep local features. Proceedings of the 2017 IEEE International Conference on Computer Vision (ICCV).

[43] An S., Zhu H., Wei D., Tsintotas K.A., Gasteratos A. (2022). Fast and incremental loop closure detection with deep features and proximity graphs. J. Field Robot..

[44] Hausler S., Garg S., Xu M., Milford M., Fischer T. Patch-NetVLAD: Multi-Scale Fusion of Locally-Global Descriptors for Place Recognition. Proceedings of the 2021 IEEE/CVF Conference on Computer Vision and Pattern Recognition (CVPR).

[45] Jin S., Dai X., Meng Q. (2023). Loop closure detection with patch-level local features and visual saliency prediction. Eng. Appl. Artif. Intell..

[46] Jin S., Chen L., Gao Y., Shen C., Sun R. (2021). Learning a deep metric: A lightweight relation network for loop closure in complex industrial scenarios. Chin. J. Electron..

